# Risk of Bleeding after Transcatheter Aortic Valve Replacement: impact of Preoperative Antithrombotic Regimens

**DOI:** 10.21470/1678-9741-2020-0538

**Published:** 2022

**Authors:** Monirah A Albabtain, Amr A Arafat, Zaid Alonazi, Hanan Aluhaydan, Mashael Alkharji, Raneem Alsaleh, Amany Alboghdadly, Mohammed AlOtaiby, Saeed AlAhmari

**Affiliations:** 1 Pharmacy Department, Prince Sultan Cardiac Center, Riyadh, Saudi Arabia.; 2 Adult Cardiac Surgery Department, Prince Sultan Cardiac Center, Riyadh, Saudi Arabia.; 3 Cardiothoracic Surgery Department, Tanta University, Tanta, Egypt.; 4 Pharmacy College, Prince Noura Bint Abdulrahman University, Riyadh, Saudi Arabia.; 5 Department of Pharmacy Practice, Batterjee Medical College, Jeddah, Saudi Arabia.; 6 Cardiology Department, Prince Sultan Cardiac Center, Riyadh, Saudi Arabia.

**Keywords:** Transcatheter Aortic Valve Replacement, Hemorrhage, Patelet Aggregation Inhibitors, Fibrinolytic Agents, Stroke, Renal Insufficiency

## Abstract

**Introduction:**

Bleeding after transcatheter aortic valve replacement (TAVR) has a negative impact on the outcome of the procedure. Risk factors for bleeding vary widely in the literature, and the impact of preoperative antithrombotic agents has not been fully established. The objectives of our study were to assess bleeding after TAVR as defined by the Valve Academic Research Consortium-2 (VARC-2), identify its risk factors, and correlate with antithrombotic treatment in addition to its effect on procedural mortality.

**Methods:**

The study included 374 patients who underwent TAVR from 2009 to 2018. We grouped the patients into four groups according to the VARC-2 definition of bleeding. Group 1 included patients without bleeding (n=265), group 2 with minor bleeding (n=22), group 3 with major bleeding (n=61), and group 4 with life-threatening bleeding (n=26). The median age was 78 (25^th^-75^th^ percentiles: 71-82), and 226 (60.4%) were male. The median EuroSCORE was 3.4 (2-6.3), and there was no difference among groups (*P*=0.886). The TAVR approach was transfemoral (90.9%), transapical (5.6%), and trans-subclavian (1.9%). Results: Predictors of bleeding were stroke (OR: 2.465; *P*=0.024) and kidney failure (OR: 2.060; *P*=0.046). Preoperative single and dual antiplatelet therapy did not increase the risk of bleeding (*P*=0.163 and 0.1, respectively). Thirty-day mortality occurred in 14 patients (3.7%), and was significantly higher in patients with life-threatening bleeding (n=8 [30.8%]; *P*<0.001). Conclusion: Bleeding after TAVR is common and can be predicted based on preprocedural comorbidities. Preprocedural antithrombotic therapy did not affect bleeding after TAVR in our population.

**Table:** Abbreviations, acronyms & symbols

AF	= Atrial fibrillation
BARC	= Bleeding Academic Research Consortium
DAPT	= Dual antiplatelet therapy
LTB	= Life-threatening bleeding
MI	= Myocardial infarction
NOAC	= Non-vitamin K antagonist oral anticoagulant
OR	= Odds ratio
PCI	= Percutaneous coronary intervention
PVD	= Peripheral vascular disease
SAPT	= Single antiplatelet therapy
SPSS	= Statistical Package for the Social Sciences
SAVR	= Surgical aortic valve replacement
TAVR	= Transcatheter aortic valve replacement
TIA	= Transient ischemic attack
VARC-2	= Valve Academic Research Consortium-2

## INTRODUCTION

Transcatheter aortic valve replacement (TAVR) can decrease 30-day and 1-year mortality compared to surgical aortic valve replacement (SAVR)^[1]^. Although TAVR has a high success rate, ischemic and bleeding complications are common, with a 5% incidence of 30-day stroke, a 17% incidence of major bleeding, and a negligible risk of 30-day myocardial infarction (MI)^[2]^.

Dual antiplatelet therapy (DAPT) is recommended for six months after TAVR^[3]^, and the current regimen of antithrombotic therapy includes a loading dose of clopidogrel before the procedure. However, in a meta-analysis, DAPT with preoperative clopidogrel loading was associated with increased bleeding after TAVR^[2,4]^.

Risk factors for bleeding after TAVR vary widely in the literature^[5,6]^. Bleeding negatively affects the clinical outcomes and has a significant impact on patients' quality of life, including total dependence on caregivers, rehospitalizations, increased length of hospital stay, cost and mortality^[7]^. Furthermore, there are conflicting reports on post-TAVR bleeding because of inconsistent definitions of bleeding used in previous studies^[8]^.

The impact of preprocedural antithrombotic therapy on post-TAVR bleeding has not been fully established^[9]^. The objectives of our study were to assess post-TAVR bleeding as defined by the Valve Academic Research Consortium-2 (VARC-2), identify its risk factors, and correlate it to antithrombotic treatment in addition to its effect on procedural mortality.

## METHODS

### Study Design

This research is a single-center retrospective cohort study of 407 consecutive patients who underwent TAVR from April 2009 to September 2018. Patients who had an aborted procedure (n=3), preprocedure use of warfarin only (n=4), or non-vitamin K antagonist oral anticoagulant (NOAC) only (n=8) and patients with missing data (n=34) were excluded. A total of 374 patients were included in the analysis.

### Data Collection, Outcome and Definitions

Clinical and procedural data and the outcomes were collected by reviewing patients' electronic medical records, and the data from pre- and post-antithrombotic regimens were recorded. We have redefined this sample using the VARC-2 tool as the most recent scoring system for bleeding complications in such patients^[10]^.

The primary outcome was to determine bleeding events after TAVR in relation to antithrombotic therapy as defined by VARC-2 bleeding categories. The secondary outcome was to estimate 30-day mortality after TAVR.

VARC-2 was updated from the previously developed VARC consensus, which has unified endpoint definitions in TAVR trials and registries and provided guidelines for standardized TAVR clinical outcome reporting^[10,11]^.

Bleeding was defined based on the VARC-2 categories as minor bleeding, major bleeding and life-threatening bleeding (LTB). Bleeding was considered LTB if it was fatal, occurred in a critical organ, caused a hypovolemic shock that required inotropes or surgical intervention, or led to a drop in hemoglobin of 5 g/dL or required blood transfusion of 4 units. Major bleeding was defined as bleeding that led to a drop in hemoglobin of 3 g/dL or required transfusion of 2-3 units. Minor bleeding was bleeding that did not meet the major and LTB criteria.

### Groups

Patients were divided according to the bleeding complication into four groups. Group 1 included patients without bleeding (n=265), group 2 had patients with minor bleeding (n=22), group 3 had patients with major bleeding (n=61), and group 4 had patients with life-threatening bleeding (n=26).

### Ethical Approval

The local Institutional Review Board approved the study (Reference number: R18010), and patient consent was waived.

### Statistical Analysis

Continuous data were presented as median with 25^th^ and 75^th^ percentiles according to normality distribution. Categorical variables were presented as frequencies and percentages. The Kruskal-Wallis test was used to determine the difference between continuous data among multiple independent groups. Post-hoc pairwise multiple comparisons were made using Dunn's tests. To compare the difference between categorical variables, Pearson's χ^2^ test or Fisher’s exact test were used as appropriate. Univariable analysis was performed on the variables in Table 1. Multivariable logistic regression analysis was performed to evaluate the independent risk factors for bleeding events. A *P*-value of <0.05 was considered statistically significant for all tests. We chose variables for multivariable analysis based on the evidence from the literature, in addition to the clinical judgment. We added the antithrombotic regimen to the model to test its effect on major and life-threatening bleeding. The model goodness-of-fit was tested using the Hosmer-Lemeshow test. All statistical analyses were performed using IBM-SPSS statistics version 21 (IBM Corp., Armonk, New York, USA).

**Table 1:** Baseline characteristics of patients. Continuous data were presented as median (25^th^ and 75^th^ percentiles) and categorical variables as numbers and percentages.

		Groups according to VARC-2 bleeding categories				
Variable	All patients (n=374)	No bleeding (n=265)	Minor bleeding (n=22)	Major bleeding (n=61)	Life-threatening bleeding (n=26)	*P*-value
Age (years)	78 (71, 82)	78 (70.5, 82.5)	78 (68.5, 81.2)	79 (74, 82)	76 (70.5, 80.8)	0.720
Male	226 (60.4)	162 (61.1)	15 (68.2)	35 (57.4)	14 (53.8)	0.725
BMI (kg/m^2^)	29.3 (25.8,29.3)	29.3 (25.9, 33.9)	28.2 (24.1, 32.7)	30.7 (27.3, 35.2)	31.8 (28.2, 35.9)	0.134
Hypertension	292 (78.1)	207 (78.1)	17 (77.3)	51 (83.6)	17 (65.4)	0.315
Diabetes mellitus	233 (62.3)	173 (65.3)	12 (54.5)	37 (60.7)	11 (42.3)	0.109
Previous MI	40 (10.6)	29 (11)	3 (13.6)	7 (11.5)	1 (3.8)	0.817
Previous TIA	4 (1.1)	1 (0.4)	0	3 (4.9)	0	0.016
Previous stroke	32 (8.6)	16 (6)	4 (18.2)	8 (13.1)	4 (15.4)	0.048
COPD	70 (18.7)	49 (18.5)	2 (9.1)	14 (23)	5 (19.2)	0.558
PVD	7 (1.9)	4 (1.5)	0	3(4.9)	0	0.242
Liver disease	15 (4)	9 (3.4)	0	2 (3.3)	4 (15.4)	0.019
Anemia*	177 (47.3)	129 (48.7)	9 (40.9)	28 (45.9)	11 (42.3)	0.833
**Previous interventions**						
Previous cardiac surgery	53 (14.2)	39 (14.7)	4 (18.2)	4 (6.6)	6 (23.1)	0.175
PCI	120 (32.2)	80 (30.2)	10 (47.6)	22 (36.1)	8(30.8)	0.359
**Laboratory values**						
Hemoglobin	12 (10.9,13.3)	12 (11,13.2)	12.4 (11.1, 13.2)	12.5 (10.7,13.6)	12.5 (11,14.4)	0.702
Platelets	237 (45,83)	237 (191,291)	220 (198,277)	257 (205,308)	257 (208,309)	0.121
INR	1.1 (1, 1.1)	1.1 (1,1.2)	1.1 (1,1.3)	1.1 (1,1.1)	1.1 (1.1,1.2)	0.334
Bilirubin	7 (5,11)	7 (5,10)	9 (6,11)	7 (5,10.5)	11.5 (8,19)	0.670
**Kidney function**						
Kidney failure**						
Moderate	173 (46.3)	115 (43.4)	13 (59.1)	30 (49.2)	15 (57.7)	0.452
Severe	103 (27.5)	76 (28.7)	4 (18.2)	16 (26.2)	7 (26.9)	
Dialysis	12 (3.2	7 (2.6)	0	4 (6.6)	1 (3.8)	0.356
**Clinical presentation**						
EuroSCORE II	3.4 (2,6.3)	3.4 (1.9,11)	3.3 (2.3, 4.7)	3.4 (1.9.6.1)	3.5 (2.4,9.2)	0.886
AF	47 (12.6)	33 (12.5)	3 (13.6)	8 (13.1)	3 (11.5)	0.995
HF within 2 weeks	67 (18.3)	42 (16.2)	4 (19)	14 (23)	7 (28)	0.352
NYHA class						
Class II	37 (9.9)	20 (7.5)	3 (13.6)	10 (16.4)	4 (15.4)	
Class III	236 (63.1)	175 (66)	12 (54.5)	34 (55.7)	15 (57.7)	0.859
Class IV	96 (25.7)	66 (24.9)	7 (31.8)	16 (26.2)	7 (26.9)	
**Echocardiography**						
LV ejection fraction (%)	55 (45,60)	55 (45,60)	55 (40,60)	55 (45,60)	55 (39,60)	0.918
LVIDd (mm)	49 (44,53)	49 (44,53)	50 (46,52)	49 (44,53)	50 (46,56)	0.743
LVIDs (mm)	33 (29,38)	32 (28,37)	34 (31,37)	32 (29,38)	36 (29,42)	0.252
PASP (mmHg)	40 (30,50)	40 (30,50)	40 (33,53)	40 (30,51)	38 (30,50)	0.984
Mean AV gradient	44.7 (38.7,54.5)	44.8 (38.1,55.3)	44.6(40.7,53)	42 (37.3, 50.7)	48.9 (40.1,60.2)	0.364
Aortic regurgitation ≥2	95 (25.4)	59 (22.3)	8 (36.4)	19 (31.1)	9 (34.6)	0.172
**Antithrombotic therapy**						
DAPT	153 (40.9)	105 (39.6)	12 (54.5)	28 (45.9)	8 (30.8)	0.307
DAPT+ warfarin	1 (0.3)	0	0	0	1 (3.8)	0.004
DAPT + NOAC	3 (0.8)	2 (0.8)	0	1 (1.6)	0	0.817
SAPT	158 (42.2)	110 (41.5)	8 (36.4)	28 (45.9)	12 (46.2)	0.833
SAPT + warfarin	11 (2.9)	9 (3.4)	2 (9.1)	0	0	0.125
SAPT + NOAC	11 (2.9)	9 (3.4)	0	0	2 (7.7)	0.190
NOAC only	9 (2.4)	7 (2.6)	0	2 (3.3)	0	0.695
Warfarin only	4 (1.1)	2 (0.8)	0	2 (3.3)	0	0.310

AF=atrial fibrillation; AV=atrioventricular; BMI=body mass index; COPD=chronic obstructive pulmonary disease; DAPT=dual antiplatelet therapy; HF=heart failure; INR=international normalization ratio; LIVDd=left ventricular internal diameter end diastole; LIVDs=left ventricular internal diameter end systole; LV=left ventricle; MI=myocardial infarction; NOAC=Non-vitamin K antagonist; NYHA=New York Heart Association; PASP=pulmonary artery systolic pressure; PCI=percutaneous coronary intervention; PVD=peripheral vascular disease; SAPT=single-antiplatelet therapy.

* History of anemia or Hgb <12 g/dL;

** Following EuroSCORE II definitions

## RESULTS

### Preoperative Characteristics

The median age of the patients was 78 (25^th^-75^th^ percentiles: 71-82), and 226 (60.4%) were male. Most of the demographic data were comparable among groups. Patients with a history stroke, transient ischemic attacks (TIA), and liver disease had significantly more major or life-threatening bleeding. There was no difference in laboratory values and clinical presentation among groups. The median EuroSCORE was 3.4 (2-6.3), and there was no difference among groups (*P*=0.886). Echocardiographic measurements were comparable among groups.

The minor bleeding events in patients using single antiplatelet therapy (SAPT) were 8 out of 158 (5%), SAPT + warfarin 2 out of 11 (18.2%), SAPT + NOAC 0 out of 11, DAPT 12 out of 153 (7.8%), and DAPT + warfarin 2 out of 11 (18.2%). Major and life-threatening bleeding events were observed in 28 (17.7%) and 12 (7.6 %) for SAPT and 28 (18.3%) and 8 (5.2%) for the DAPT group of patients, respectively (Table 1).

### Operative and Postoperative Outcomes

The TAVR approach was transfemoral (n=345; 90.9%), transapical (n=21; 5.6%), and trans-subclavian (n=7; 1.9%). The procedure was aborted in three patients because of small femoral arteries, failure to dilate the aortic annulus, and large left ventricular pseudoaneurysm.

There was no difference in valve types and concomitant percutaneous coronary intervention (PCI) among groups. Postoperative stroke, need for new dialysis, new atrial fibrillation (AF) and major vascular complications were significantly higher in patients with LTB. Coronary care unit stay was longer in patients with major bleeding compared to patients without bleeding (*P*=0.003) and in LTB compared to patients without bleeding (*P*=0.042). The hospital stay was longer in patients with major bleeding than without bleeding (*P*=0.003) and in LTB compared to patients without bleeding (*P*=0.011). The lowest hemoglobin was reported in patients with LTB (*P*<0.001) (Table 2).

**Table 2:** Operative and post-procedure characteristics and events.

		Groups according to VARC-2 bleeding categories				
Variable	All patients (n=374)	No bleeding (n=265)	Minor bleeding (n=22)	Major bleeding (n=61)	Life-threatening bleeding (n=26)	*P*-value
Aborted procedure	3 (0.8)	1 (0.4)	0	1 (1.6)	1 (3.8)	0.227
Aortic valve-in-valve						
Concomitant PCI	19 (5.1)	11 (4.2)	3 (13.6)	3 (4.9)	2 (7.7)	0.242
**Approach**						
Transapical	21 (5.6)	18 (6.8)	0	2 (3.3)	1 (3.8)	0.426
Transfemoral	345 (92.2)	241 (90.9)	22 (100)	58 (95.1)	24 (92.3)	0.367
Trans-subclavian	7 (1.9)	5 (1.9)	0	1 (1.6)	1 (3.8)	0.804
Transaortic	1 (0.3)	1 (0.4)	0	0	0	0.938
**THV type**						
Balloon-expandable	124 (33.2)	82 (30.9)	5 (22.7)	25 (41)	12 (46.2)	0.149
Self-expandable	247 (66)	182 (68.7)	17 (77.3)	35 (57.4)	13 (50)	0.069
CCU stay (days)	2 (1,4)	2 (1, 3)	3 (1, 5)	3 (1, 6)	4 (1, 13)	<0.001
In-hospital stay (days)	5 (3,7)	5 (3, 6)	5 (3, 5)	6 (4, 12)	10 (3, 24)	<0.001
**Post-procedure events**						
30-day mortality	14 (3.7)	5 (1.9)	0	1 (1.6)	8 (30.8)	<0.001
Coronary occlusion	5 (1.3)	0	1(4.5)	0	4 (15.4)	<0.001
Stroke	7 (1.9)	0	0	4 (6.6)	3 (11.5)	<0.001
Need for dialysis	10 (2.7)	4 (1.5)	0	2 (3.3)	4 (15.4)	<0.001
Major vascular complications^§^	66 (17.6)	1 (0.4)	4 (18.2)	39 (63.9)	22 (84.6)	<0.001
Minor vascular complications^§^	38 (10.2)	0	18 (81.8)	19 (31.1)	1 (3.8)	<0.001
Need for PPM	78 (20.9)	61 (23)	3 (13.6)	11 (18)	3 (11.5)	0.375
New AF	13 (3.5)	7 (2.6)	0	2 (3.3)	4 (15.4)	0.006
Lowest Hb	10.1 (9, 11.3)	10.4 (9.2, 11.6)	10.6 (9.8, 11.7)	9.4 (8.4, 10.7)	8.6 (8, 9.6)	
Transfusion ≥2 units	33 (8.8)	7 (2.7)	0	11 (18)	15 (57.7)	<0.001
Paravalvular leak (2+)	41 (11)	29 (10.9)	3 (13.6)	4 (6.6)	5 (19.2)	0.363

AF=atrial fibrillation; CCU=coronary care unit; Hb=hemoglobin; PCI=percutaneous coronary intervention; PPM=permanent pacemaker; THV=transcatheter heart valve.

§ Following VARC-2 definitions

Thirty-day mortality occurred in 14 patients (3.7%), and was significantly higher in patients with LTB (n=8 [30.8%]; *P*<0.001).

### Predictors of Postoperative Bleeding

Predictors of bleeding were history of stroke (OR: 2.465 (1.127-5.392); P=0.024), and kidney failure (OR: 2.060 (1.013-4.190); *P*=0.046). Preoperative single and dual antiplatelet therapy did not increase the risk of bleeding (*P*=0.163 and 0.1, respectively) (Table 3).

**Table 3:** Multivariable analysis of predictors of bleeding.

Binary logistic regression for major and life-threatening bleeding events		
Variable	OR, 95% CI	*P*-value
Age	0.995 (0.963-1.028)	0.758
Male	0.695 (0.415-1.162)	0.165
Kidney failure	2.060 (1.013-4.190)	0.046
Liver disease	2.356 (0.796-6.973)	0.122
Previous stroke	2.465 (1.127-5.392)	0.024
Dual antiplatelet	1.745 (0.798-3.816)	0.163
Single antiplatelet	1.906 (0.884-4.109)	0.100

Hosmer-Lemeshow goodness-of-fit (χ^2^=6.457; df=8; *P*=0.716)

Interaction between antithrombotic regimen and kidney failure, liver disease and stroke was tested, and there was no significance.

## DISCUSSION

Early bleeding is a common complication after TAVR, affecting 30% to 70% of TAVR patients^[3,12]^. This high incidence is probably a result of the unique characteristics of TAVR patients. Patients who underwent TAVR are mostly older people who, due to advanced age, concomitant anemia, kidney failure and low body mass, have a high risk of bleeding^[7,12,13]^.

We carried out this study to evaluate bleeding complications after TAVR and to identify its risk factors. Additionally, we investigated the effect of preoperative antithrombotic drugs on postoperative bleeding. We have used the recent VARC-2 endpoint definitions to report clinical outcomes after TAVR^[10]^. The VARC definitions were recently updated and published as VARC-2^[10]^. VARC-2 contains the initial definitions and recognizes the agreement of the Bleeding Academic Research Consortium (BARC). In this way, it advanced the standardization of the endpoint definitions for clinical assessment of TAVR. By using standardized definitions, the outcomes after TAVR can be compared and improved among different centers.

The incidences of major bleeding and LTB after TAVR in this study were 16.3% and 6.6%, respectively, which is still in the percentage range previously reported, 2% to 40%^[7,14-16]^. In a meta-analysis of large TAVR registries, the overall bleeding rate ranges from 4.6% in the university hospital Zurich TAVI Registry to 43.3% in the PRAGMATIC Multicenter Study^[17]^. The rates of bleeding events post-TAVR vary widely in the literature, which can be attributed to the use of different bleeding definitions. This issue was managed later with the establishment of VARC and updated VARC-2 definitions of post-TAVR bleeding^[18]^.

Several risk factors for bleeding were reported; in our study, we found that previous stroke and kidney failure were the only independent factors for post-TAVR bleeding. Transapical access and preexisting atrial fibrillation were found as independent predictors of post-TAVR bleeding in other studies^[18]^. Access site did not affect bleeding in our study because the main access site we used was transfemoral, and few patients had a transapical approach. Transapical access is a more invasive approach that explained the higher bleeding rates with this approach in other series.

Previous peripheral vascular disease (PVD) and preexisting anemia significantly increased the incidence of bleeding in a meta-analysis^[18]^. However, the study heterogeneity was exceptionally high, and hemoglobin and platelets did not appear to be confounding factors for the meta-analysis findings. In our study, PVD and anemia did not have a predictive effect on post-TAVR bleeding. Our regression showed that hemoglobin and platelet levels are not predictors for major and life-threatening bleeding events, consistent with previous findings^[7]^.

Despite the high prevalence of TAVR bleeding, the influence of preprocedural antithrombotic regimens on bleeding has not been fully established^[12]^. Antithrombotics (antiplatelet and anticoagulants) are a common practice in such populations, especially with coexisting AF and/or acute coronary syndrome^[19]^. The antithrombotic strategy after TAVR was investigated in several trials, such as ATLANTIS^[20]^ and ENVISAGE^[21]^.

Dual antiplatelet therapy has increased the risk of post-TAVR bleeding by 410%; clopidogrel alone increased the risk by 470%^[3,12]^. In our study, the effect of pre-TAVR antithrombotic treatment on post-TAVR bleeding events was not statistically significant, and patients who received pre-TAVR SAPT or DAPT were not at increased risk of bleeding. Adding warfarin or NOACs to SAPT or DAPT did not increase the risk of bleeding, but this can be attributed to the low number of these patients. The effect of antithrombotic drugs did not change in the presence of stroke, anemia, kidney failure or liver disease.

### Effect of TAVR-Associated Bleeding on The Incidence of 30-Day Mortality

Vascular complications and major bleeding considerably increased the risk of 30-day and 1-year mortality irrespective of the bleeding etiology^[22,23]^.

In this study, the 30-day mortality was reported in 14 patients (3.7%). It was 1 and 8% for major bleeding and LTB, respectively. This rate is lower than that reported in a meta-analysis of 12 studies, which reported that early bleeding and vascular complications accounted for nearly 18% of deaths in the first 30 days after the procedure^[18]^. In this meta-analysis, 150 patients observed that 16% of the initial vascular complications were significantly related to bleeding events. From the results of the SOURCE registry, vascular complications were observed in 22.9% of patients and markedly increased early mortality after transapical implantation^[12,24]^. The low mortality reported in our cohort could be related to the lower EuroSCORE in our population. The 30-day mortality was higher in patients with LTB (30.8%) *versus* major bleeding or no bleeding (*P*<0.001).

Our study results indicated that bleeding is common after TAVR, and 23% of patients had major or life-threatening bleeding. History of stroke and kidney failure predicted major or more severe bleeding. These patients may benefit from proper preoperative optimization of their medical condition (such as increasing Hb level) to decrease bleeding and, consequently, mortality.

### Limitation of the Study

The main limitation of our study is the retrospective design with its inherent referral and selection biases; however, this study design is accepted for analyzing the complications of the procedure since it presents a real-life experience. Our study included several antithrombotic regimens, and some of them had a very low frequency; therefore, their true effect may not be properly estimated. Larger studies and a longer follow-up period are recommended. The study is a single-center experience, and the generalization of results may be an issue; however, our population is understudied in the literature.

## CONCLUSION

In this study, post-TAVR bleeding was common and could be predicted based on preprocedural comorbidities. Preoperative antithrombotic therapy did not affect post-TAVR bleeding in our population.

## References

[1] Takagi H, Hari Y, Nakashima K, Kuno T, Ando T, ALICE (All-Literature Investigation of Cardiovascular
Evidence) Group (2020). Mortality after transcatheter versus surgical aortic valve
replacement: an updated meta-analysis of randomised trials. Neth Heart J.

[2] Gandhi S, Schwalm JD, Velianou JL, Natarajan MK, Farkouh ME (2015). Comparison of dual-antiplatelet therapy to mono-antiplatelet
therapy after transcatheter aortic valve implantation: systematic review and
meta-analysis. Can J Cardiol.

[3] Czerwińska-Jelonkiewicz K, Zembala M, Dąbrowski M, Witkowski A, Ochała A, Kochman J (2017). Can TAVI patients receive aspirin monotherapy as patients after
surgical aortic bioprosthesis implantation? Data from the Polish registry -
POL-TAVI. Int J Cardiol.

[4] Vavuranakis M, Kalogeras K, Kolokathis AM, Vrachatis D, Magkoutis N, Siasos G (2018). Antithrombotic therapy in TAVI. J Geriatr Cardiol.

[5] Konigstein M, Ben-Assa E, Banai S, Shacham Y, Ziv-Baran T, Abramowitz Y (2015). Periprocedural bleeding, acute kidney injury, and long-term
mortality after transcatheter aortic valve implantation. Can J Cardiol.

[6] Leclercq F, Akodad M, Macia JC, Gandet T, Lattuca B, Schmutz L (2015). Vascular complications and bleeding after transfemoral
transcatheter aortic valve implantation performed through open surgical
access. Am J Cardiol.

[7] Kochman J, Rymuza B, Huczek Z, Kołtowski ł, ścisło P, Wilimski R (2016). Incidence, predictors and impact of severe periprocedural
bleeding according to VARC-2 criteria on 1-year clinical outcomes in
patients after transcatheter aortic valve implantation. Int Heart J.

[8] Pilgrim T, Stortecky S, Luterbacher F, Windecker S, Wenaweser P (2013). Transcatheter aortic valve implantation and bleeding: incidence,
predictors and prognosis. J Thromb Thrombolysis.

[9] Nijenhuis VJ, Brouwer J, Søndergaard L, Collet JP, Grove EL, Ten Berg JM (2019). Antithrombotic therapy in patients undergoing transcatheter
aortic valve implantation. Heart.

[10] Kappetein AP, Head SJ, Généreux P, Piazza N, van Mieghem NM, Blackstone EH (2013). Updated standardized endpoint definitions for transcatheter
aortic valve implantation: the valve academic research consortium-2
consensus document. J Thorac Cardiovasc Surg.

[11] Kappetein AP, Head SJ, Généreux P, Piazza N, van Mieghem NM, Blackstone EH (2012). Updated standardized endpoint definitions for transcatheter
aortic valve implantation: the valve academic research consortium-2
consensus document. J Am Coll Cardiol.

[12] Czerwińska-Jelonkiewicz K, Witkowski A, Dąbrowski M, Banaszewski M, Księżycka-Majczyńska E, Chmielak Z (2013). Antithrombotic therapy - predictor of early and long-term
bleeding complications after transcatheter aortic valve
implantation. Arch Med Sci.

[13] Huczek Z, Kochman J, Kowara MK, Wilimski R, Scislo P, Scibisz A (2015). Baseline platelet indices and bleeding after transcatheter aortic
valve implantation. Blood Coagul Fibrinolysis.

[14] Buchanan GL, Chieffo A, Montorfano M, Maisano F, Latib A, Godino C (2011). The role of sex on VARC outcomes following transcatheter aortic
valve implantation with both Edwards SAPIEN™ and medtronic corevalve
revalving system® devices: the Milan registry. EuroIntervention.

[15] Petronio AS, Giannini C, De Carlo M, Bedogni F, Colombo A, Tamburino C (2016). Anaesthetic management of transcatheter aortic valve
implantation: results from the Italian corevalve registry. EuroIntervention.

[16] Chieffo A, Petronio AS, Mehilli J, Chandrasekhar J, Sartori S, Lefèvre T (2016). Acute and 30-day outcomes in women after TAVR: results from the
WIN-TAVI (women's international transcatheter aortic valve implantation)
real-world registry. JACC Cardiovasc Interv.

[17] Chieffo A, Van Mieghem NM, Tchetche D, Dumonteil N, Giustino G, Van der Boon RM (2015). Impact of mixed aortic valve stenosis on VARC-2 outcomes and
postprocedural aortic regurgitation in patients undergoing transcatheter
aortic valve implantation: results from the international multicentric study
PRAGMATIC (pooled Rotterdam-Milan-Toulouse in collaboration). Catheter Cardiovasc Interv.

[18] Wang J, Yu W, Jin Q, Li Y, Liu N, Hou X (2017). Risk factors for post-TAVI bleeding according to the VARC-2
bleeding definition and effect of the bleeding on short-term mortality: a
meta-analysis. Can J Cardiol.

[19] Généreux P, Cohen DJ, Mack M, Rodes-Cabau J, Yadav M, Xu K (2014). Incidence, predictors, and prognostic impact of late bleeding
complications after transcatheter aortic valve replacement. J Am Coll Cardiol.

[20] Collet JP, Berti S, Cequier A, Van Belle E, Lefevre T, Leprince P (2018). Oral anti-Xa anticoagulation after trans-aortic valve
implantation for aortic stenosis: the randomized ATLANTIS
trial. Am Heart J.

[21] Van Mieghem NM, Unverdorben M, Valgimigli M, Mehran R, Boersma E, Baber U (2018). Edoxaban versus standard of care and their effects on clinical
outcomes in patients having undergone transcatheter aortic valve
implantation in atrial fibrillation-rationale and design of the
ENVISAGE-TAVI AF trial. Am Heart J.

[22] Stortecky S, Stefanini GG, Pilgrim T, Heg D, Praz F, Luterbacher F (2015). Validation of the valve academic research consortium bleeding
definition in patients with severe aortic stenosis undergoing transcatheter
aortic valve implantation. J Am Heart Assoc.

[23] Généreux P, Cohen DJ, Williams MR, Mack M, Kodali SK, Svensson LG (2014). Bleeding complications after surgical aortic valve replacement
compared with transcatheter aortic valve replacement: insights from the
PARTNER I trial (placement of aortic transcatheter valve). J Am Coll Cardiol.

[24] Al-Atassi T, Thourani VH (2017). Early results of the SOURCE 3 registry: a source of encouragement
with some caveats. Circulation.

